# Targeting Ferroptosis against Ischemia/Reperfusion Cardiac Injury

**DOI:** 10.3390/antiox10050667

**Published:** 2021-04-25

**Authors:** José Lillo-Moya, Catalina Rojas-Solé, Diego Muñoz-Salamanca, Emiliano Panieri, Luciano Saso, Ramón Rodrigo

**Affiliations:** 1 Molecular and Clinical Pharmacology Program, Institute of Biomedical Sciences, Faculty of Medicine, University of Chile, Santiago 8380000, Chile; joselillo@ug.uchile.cl (J.L.-M.); catalinarojass@ug.uchile.cl (C.R.-S.); diego.munoz.s@ug.uchile.cl (D.M.-S.); 2 Department of Physiology and Pharmacology “Vittorio Erspamer“, Faculty of Pharmacy and Medicine Sapienza University, P.le Aldo Moro 5, 00185 Rome, Italy; emiliano.panieri@isprambiente.it (E.P.); luciano.saso@uniroma1.it (L.S.)

**Keywords:** cardioprotection, oxidative stress, ischemia, reperfusion, ferroptosis, liproxstatin-1

## Abstract

Ischemic heart disease is a leading cause of death worldwide. Primarily, ischemia causes decreased oxygen supply, resulting in damage of the cardiac tissue. Naturally, reoxygenation has been recognized as the treatment of choice to recover blood flow through primary percutaneous coronary intervention. This treatment is the gold standard therapy to restore blood flow, but paradoxically it can also induce tissue injury. A number of different studies in animal models of acute myocardial infarction (AMI) suggest that ischemia-reperfusion injury (IRI) accounts for up to 50% of the final myocardial infarct size. Oxidative stress plays a critical role in the pathological process. Iron is an essential mineral required for a variety of vital biological functions but also has potentially toxic effects. A detrimental process induced by free iron is ferroptosis, a non-apoptotic type of programmed cell death. Accordingly, efforts to prevent ferroptosis in pathological settings have focused on the use of radical trapping antioxidants (RTAs), such as liproxstatin-1 (Lip-1). Hence, it is necessary to develop novel strategies to prevent cardiac IRI, thus improving the clinical outcome in patients with ischemic heart disease. The present review analyses the role of ferroptosis inhibition to prevent heart IRI, with special reference to Lip-1 as a promising drug in this clinicopathological context.

## 1. Introduction

Cardiovascular diseases are major causes of death and disability, reaching 17.9 million deaths in 2016 [1], among which ischemic heart disease is the leading cause of death along with stroke, respectively accounting for 16% and 11% of the total deaths worldwide [2]. This represents an estimated total cost of 196,000 million euros per year in cardiovascular disease in Europe, approximately 54% of the total investment in health, and corresponds to a 24% loss of productivity [3]. Ischemic heart disease occurs through thrombotic complications derived from atherosclerotic plaques in the coronary arteries, producing a series of biochemical and metabolic changes that eventually lead to the death of cardiomyocytes. This cell death is further exacerbated when the occlusion of the coronary arteries is complete, generating an acute myocardial infarction (AMI). Here, the coronary microcirculation is significantly reduced, affecting the thickness of heart walls along with producing structural and functional disability, scarring and adverse remodeling [4,5]. It has been shown that the most effective interventions to reduce the infarct size and improve the clinical outcome are thrombolytic therapy and percutaneous coronary angioplasty aimed at restoring blood flow. However, paradoxically the latter can induce the death of cardiomyocytes and increase the infarct size, thereby reducing its beneficial effects. This phenomenon is known as ischemia-reperfusion injury (IRI), which can be responsible for up to 50% of the final infarct size [6]. Currently, there is no available therapy to prevent heart IRI, which makes it necessary to delve into the pathophysiology of this clinical model. A number of studies have demonstrated that oxidative and nitrosative stress are leading causes of IRI, prompted by increased production of reactive oxygen species (ROS) and reactive nitrogen species (RNS) in the process of ischemia followed by reperfusion. In addition, another important contribution derives from the deregulation of iron homeostasis, which causes an increase in the myocardial intracellular free iron. This metal ion increases the production of hydroxyl radical (^•^OH) via the Fenton reaction. Finally, these reactive species increase cellular injury through the attack on biomolecules such as lipids, DNA and proteins, besides the activation of various cell death pathways, such as apoptosis, necrosis, pyroptosis, and ferroptosis [7]. Ferroptosis has been studied in different ischemia/reperfusion (I/R) models, concluding that it is the most important driver of the final infarct size [8,9]. However, currently there is no therapy to prevent IRI and additional studies are needed in this clinical model. Thus, the present review describes the cellular and molecular mechanisms related to oxidative stress induced by ischemia-reperfusion and deregulation of iron homeostasis, focused on the essential role of inhibition of ferroptosis to prevent IRI and the role of liproxstatin-1 as a promising drug in the treatment of this clinicopathological condition.

## 2. Oxidative Stress

### 2.1. Production of Reactive Oxygen Species and Reactive Nitrogen Species

Oxidative stress is a pathogenetic mechanism based on an imbalance between production of ROS over the antioxidant defense system in the body and the loss of control over redox signaling events within the cells [10,11]. ROS are formed either by enzymatic or non-enzymatic production in mammalian cells as intermediates of the reduction of oxygen molecules to water during cellular metabolism, such as superoxide (O_2_^•−^), hydrogen peroxide (H_2_O_2_) and hydroxyl radical (^•^OH) [12]. RNS include nitrogen dioxide radical (NOO^•^), peroxynitrite anion (ONOO^−^) and nitric oxide radical (NO^•^). NO^•^ is generated in biological tissues by specific nitric oxide synthases (NOSs), which metabolize arginine to citrulline with the formation of NO^•^. Additionally, in some situations such as inflammatory processes, formation of O_2_^•−^ and NO^•^ occurs, thus triggering the production of ONOO^−^, a potent oxidizing molecule that can cause DNA fragmentation or lipid oxidation [13,14]. The production of ROS occurs mostly within the mitochondria of a cell, where more than 10 ROS production sites have been described in the isolated rat skeletal muscle mitochondria, which correspond to sites O_f_, P_f_, B_f_, A_f_ ubicated in the 2-oxoacid dehydrogenases complexes, sites I_f_ and I_Q_ ubicated in complex I, III_Qo_ ubicated in complex III and finally the sites II_f_, G_Q_, E_f_ and D_Q_ are linked to the Q-dependet dehydrogenases in the QH_2_/Q isopotential pool [15]. These sites generate ROS in different proportions. More specifically it is believed that I_f_, III_Qo_ and G_Q_ mainly generate superoxide, II_f_ generates superoxide and hydrogen peroxide and O_f_, P_f_, B_f_ and A_f_ generate predominantly superoxide or a mixture of superoxide and hydrogen peroxide, although additional studies are required to substantiate this evidence. The contribution of sites E_f_ and D_Q_ is still a matter of debate [15]. Furthermore, the site that generates more ROS in the mitochondria is commonly associated with complex I, while this actually corresponds to 2-oxoglutarate dehydrogenase complex (Of), which generates eight times more superoxide/hydrogen peroxide than site I_f_ in skeletal muscle mitochondria under optimum conditions [16]. Nevertheless, in cardiac tissue exposed to I/R, there are other ROS sources, such as xanthine oxidase (XO) in endothelial cells, reduced nicotinamide adenine dinucleotide phosphate (NADPH) oxidase (NADPHox) in neutrophils, mitochondrial electron transport chain (mETC), uncoupled NOS (uncNOS), cytochrome P450, lipoxygenase (LOX), cyclooxygenase and monoamine oxidase [17,18]. Additionally, non-enzymatic generation of ROS occurs in the presence of metal ions, such as free iron that exists in the labile iron pool (LIP) and can directly generate highly toxic hydroxyl radicals (OH^•^) via Fenton and Haber–Weiss reactions [7] (Reactions 1 and 2).

In the Fenton reaction, ferrous iron reacts with hydrogen peroxide forming the products ferric iron and hydroxyl radical in a non-enzymatic process.

Reaction 1             Fe^2+^    +   H_2_O_2_  →   Fe^3+^ + ^−^OH + ^•^OH

In the Haber–Weiss reaction, iron catalyzes the reaction to increase the production of hydroxyl radical:

Reaction 2             O_2_^•−^    +   H_2_O_2_  →  O_2_ + ^−^OH + ^•^OH

The hydroxyl radical has a high reactivity and low specificity, making it a very dangerous radical, capable of attacking all classes of biological molecules to a higher rate than O_2_^•−^ [7,19]. This becomes extremely relevant in cardiac tissue exposed to I/R. Indeed, during this condition, there is an increase in LIP, which can produce more ^•^OH via the Fenton reaction to inflict damage on DNA, proteins and lipids through DNA oxidation, protein carbonylation and lipid peroxidation [7,20]. Furthermore, the cellular effects of ROS are partially mediated by nuclear factor kappa B (NF-κB) activation, where ROS induces phosphorylation of the inhibitory cofactor IκB, triggering NF-κB to translocate into the nucleus, bind the DNA response element, and promote the transcription of genes involved in the inflammatory and pro-fibrotic response, being the major mediator of cytokine effects in the heart. In addition, NF-κB regulates cardiac gene expression, which is induced by multiple signal transduction cascades in a variety of physiological and pathophysiological states [21].

### 2.2. Antioxidant Systems

The antioxidant systems regulate redox homeostasis by controlling the intracellular ROS levels and their interaction with the biological constituents during normal cellular metabolism and pathophysiological states. This multilayered system comprises antioxidant enzymes, such as superoxide dismutase (SOD), glutathione peroxidase (GPX) and catalase (CAT), which are the first line of cellular defense against oxidative injury in the heart as well as most tissues. As a complement to their activity, non-enzymatic antioxidants are also present, which include a variety of molecules such as vitamin C or ascorbic acid (AA), vitamin E or α-Tocopherol (α-TOH), reduced glutathione (GSH), carotenoids, flavonoids, polyphenols and other exogenous and endogenous antioxidants [21,22,23]. There are many mechanisms whereby antioxidants may act [22,24], such as:

- Scavenging of ROS or their precursors.
- Inhibiting ROS production
- Attenuating the catalysis of ROS generation via chelating metal ions
- Enhancing endogenous antioxidant generation
- Repairing the oxidative damage inflicted on the macromolecules
- Reducing apoptotic cell death by up-regulating the anti-death gene Bcl-2.

For example, SOD can reduce O_2_^•−^ to H_2_O_2_, a molecule that is relatively stable in the absence of metal ions, but that is rapidly converted to water by CAT and GPX. These enzymes are encoded by several housekeeping genes largely controlled by the nuclear factor-erythroid 2-related factor 2 (Nrf2) transcription factor, which has been demostrated as a pre-conditionig therapeutic target to reduce IRI in isolated rabbit heart [25]. Exposure to oxidative or electrophilic stress impairs the recognition of Nrf2 by its negative regulator Kelch-like ECH-associated protein 1 (Keap 1), preventing its proteasomal degradation. As a consequence, Nrf2 can translocate into the nucleus and bind to specific DNA sequences known as antioxidant response elements (ARE) located in the promoter region of target genes, thus enhancing their expression. Of relevance, the increased expression of antioxidant enzymes protects tissues from oxidative stress and elicits a cardioprotective effect for myocardial reperfusion [21,26]. The enzyme heme-oxygenase-1 (HO-1) is involved in the oxidative catabolism of the heme group, generating biliverdin, carbon monoxide and iron as byproducts. HO-1 has antioxidant properties not only for its activity itself, but also for the products that are generated during heme metabolism, as in the case of biliverdin. HO-1 expression increases considerably in the presence of hypoxia [27], but it is not clear whether HO-1 induction is beneficial or detrimental due to the increase in the free iron after its enzymatic activity, which enhances oxidative stress despite the antioxidant activity of HO-1.

## 3. Pathophysiology of Myocardial I/R Injury

### 3.1. Ischemia

**Figure 1:**
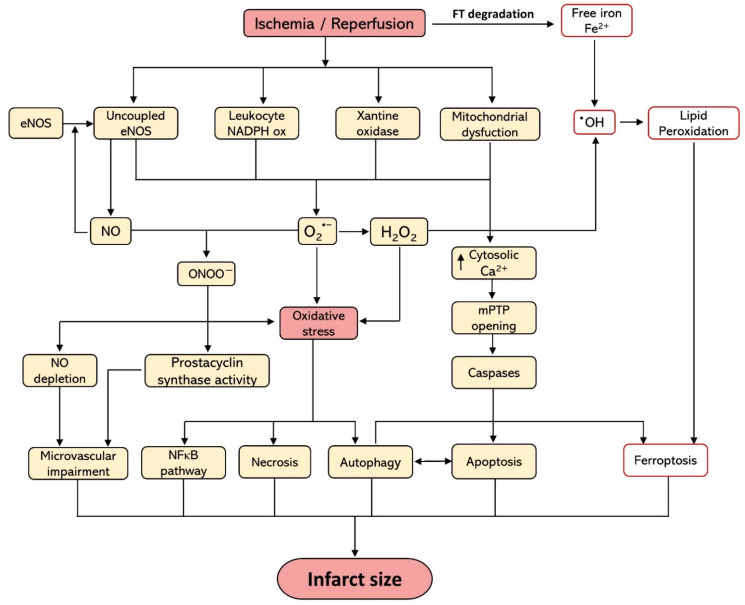
Schematic diagram of the main contributory oxidative stress-related factors involved in the pathophysiology of myocardial damage due to ischemia-reperfusion. eNOS, endothelial nitric oxide synthase; Fe^2+^, ferrous iron; FT, ferritin; H_2_O_2_: hydrogen peroxide; mPTP, mitochondrial permeability transition pore; NADPH ox, reduced nicotine adenine dinucleotide phosphate oxidase; NO, nitric oxide; O_2_^•−^, superoxide radical anion; ^•^OH, hydroxyl radical; ONOO^−^, peroxynitrite anion.

During ischemia, there is a decrease in ATP production as a result of the decrease in the supply of oxygen in the ETC. This is accompanied by a shift in cellular respiration triggering an accumulation of lactic acid [28,29] and reducing the activity of the Krebs cycle, which makes it difficult to eliminate CO_2_ [18]. This causes a series of changes in intracellular ionic homeostasis, such as a decrease in pH and ATP reserves, and an increase in intracellular Na^+^, which is due to activation of the Na^+^/H^+^ exchanger by the decrease in pH and the lack of substrate for the Na^+^/K^+^ pump that prevents the restoration of Na^+^ concentrations. The accumulation of intracellular Na^+^ forces the Ca^2+^/Na^+^ exchanger to work in the reverse mode to generate an efflux of Na^+^ to the extracellular space paralleled by an increase in the intracellular Ca^2+^ [30]. This extra Ca^2+^ cannot be captured by the sarcoplasmic reticulum because ATP is depleted and it is necessary for the function of the Ca^2+^-ATP pump [31]. The increase in Ca^2+^ induces the conversion of the enzyme xanthine dehydrogenase to XO in endothelial cells, which are capable of producing O_2_^•−^ anion and H_2_O_2_ from O_2_ [32], which markedly contribute to the induction of oxidative stress. Finally, it should be mentioned that the mitochondrial permeability transition pore (mPTP), a membrane channel located in the inner membrane of the mitochondria that can induce the uncoupling of oxidative phosphorylation when it opens, is inhibited by intracellular acidic pH (Figure 1) [33].

### 3.2. Reperfusion

During reperfusion, blood flow is restored and this produces reoxygenation of the myocardial tissue. It has been found that during the first minutes of reperfusion there is a burst in ROS production [34], which would be one of the bases of IRI due to oxidative stress. The overoxidation of lipids, DNA and proteins increases the damage and finally leads to cell death [35].

Several studies have described different sources of ROS in cardiac IRI, such as the mitochondria, NADPHox, XO and uncNOS. During cardiac IRI, mitochondria are considered as a major source of ROS, and undergo alterations of their structure and function after the injury. The alterations in the mitochondria have been associated with the ischemic processes, which subsequently causes the burst of ROS during reperfusion, mainly from the complex I and III [36]. NADPHox is an enzyme that specifically produces ROS, either in pathological or physiological conditions. In IRI, different enzymatic isoforms have been studied in knockout mice and wild-type controls, suggesting that NAPHox 1 and 2 mediated the oxidative damage during the heart reperfusion. However, in a Langendorff model, the authors observed that after reperfusion, there was a decrease in oxidative stress markers within sections of infarcted hearts in NADPHox 2 deficiency but not in NADPHox 1 deficiency [37]. Furthermore, in the study of Dulilio et al., the phagocytic NAPDHox 2 is identified as the primary source of ROS during cardiac IRI in the dog [38], suggesting that this isoform is an important therapeutic target in cardiac IRI.

Nitric oxide is formed from the conversion of L-arginine to L-citruline by NOS, but during cardiac ischemia, tetrahydrobiopterin, a cofactor of NOS, is oxidized, causing NOS to produce large amounts of superoxide during reperfusion instead of nitric oxide [35,39]. Finally, XO has been reported to play a minor role in ROS generation in the human heart during ischemia [40] due to controversy in the quantity and activity of the enzyme, making it a non-relevant therapeutic target in human myocardial IRI, in contrast to what occurs in other organs like the gastrointestinal tract or liver [17,32,41].

During a burst of oxidative stress, the intracellular acidic pH is restored to physiological values, which possibly causes mPTP opening by removing the inhibitory effect of acid pH [18,42,43]. A study indicates that reperfusion of the ischemic heart causes mitochondrial Ca^2+^ overload and creates an intracellular environment ideally suited for the opening of the mPTP. However, while this pore may remain closed during ischemia, it can be opened when pH recovery occurs [44]. This opening produces the uncoupling of oxidative phosphorylation and therefore decreases the ATP availability within the cell, which can subsequenly induce cell death through the mitochondrial pathway [45].

On the other hand, a study shows that a temporary decrease in pH during reperfusion causes a smaller infarct size in dogs [46]. The exchange of Na^+^ by the Na^+^/H^+^ exchanger activates the exchange of Na^+^/Ca^2+^. This process mediates the overload of Ca^2+^ during reperfusion. Moreover, temporary acidosis during the first minutes of reperfusion quickly recovers blood flow, which can sufficiently oxygenate the myocardium and may be beneficial to the attenuation of infarct size, and is believed to be cardio protective against IRI.

Inhibiting the Na^+^/H^+^ exchanger-1 (NHE-1) is involved in the regulation of transcriptional factors responsible for mitochondrial function. The effect of inhibitors shows beneficial effects in relation to the post-infarction myocardium in terms of morphological aspects and heart failure. An improvement in mitochondrial respiratory function and a prevention of mPTP opening were observed due to the anti-remodeling effect produced by the NHE-1 inhibitor. Another effect is downregulation of mitochondrial transcription factors induced by postinfarction remodeling. The improvement of mitochondrial function and the downregulation of hypertrophic marker gene expression is highly correlated with the transcriptional protection that regulates mitochondrial biogenesis [47].

However, the role of the molecular identity of mPTP is still a matter of discussion. Studies showed that proteins such as adenine nucleotide translocase (ANT) and FOF1-ATP synthase are proposed as the main potential candidate mPTP components, but the capacity of inducing cell death remains unclear [48,49]. ANT participates in cell death, but its cytotoxic effects are independent of mPTP, suggesting an alternative mechanism of ROS-dependent upregulation and activation of Bax (pro-death protein). Indeed, an ANT variant, ANT1, induces cell death in cardiomyocytes and was localized in the mitochondria of the myocytes. In addition, ANT1 increases in cell membrane failure and induces a loss of mitochondrial potential, but this change is not necessarily related to mPTP. These changes generated in the mitochondria, elicited a small but significant rise in ROS production and is a mechanism by which ANT1 induces even 2-fold Bax expression and cell death [48]. On the other hand, the ATP synthases are multiprotein complexes found in the energy-transducing membranes with a unique role, particularly in the mitochondrial inner membranes. FOF1-ATP synthase is an important enzyme that provides cellular energy in the form of ATP [50]. This protein can be regulated by the action of activators and inhibitors in apoptotic cell death conditions, generating a change in electrical potential around FOF1-ATP synthase. A recent study hypothesized that subunits of FOF1-ATP synthase are recycled during induction of apoptosis and thus constitute the elusive permeability transition pore/mitochondrial megachannel. However, due to several combinations of the ATP synthase subunits that could build the pore, the role of ATP synthase in the formation of the permeability transition pore/mitochondrial megachannel is more difficult [51].

## 4. Iron Homeostasis

Cardiomyocytes are vulnerable to iron deficiency, since they require huge amounts of energy and thus iron-containing mitochondrial enzymes to ensure their proper function. On the other hand, cardiomyocytes are poorly protected from iron overload because iron proteins are not directly related to the amount of iron in the body [52]. Iron importing, sequestering and exporting proteins are responsible for the proper maintenance of iron levels in cardiomyocytes. Ferritin (FT) is the main iron storage protein in a non-toxic and readily available form. Ferroxidases catalyze the conversion of Fe^2+^ to Fe^3+^ and the latter binds to transferrin (TF) for transport. However, when the TF is saturated, LIP remains and is engaged in the Fenton reaction, generating ^•^OH [53]. This radical is an important iron charge indicator and is highly reactive, thus leading to oxidative stress in various cell lines [7] and may be related to the magnitude of the infarct size (Figure 1).

### 4.1. Intracellular Iron Regulation

Several iron uptake transport systems exist in the heart, such as transferrin receptor (TfR1), divalent metal transporter (DMT1), and L-type (LTCC) or T-type (TTCC) voltage-dependent Ca^2+^ channels. Normally, the amount of iron in the human body ranges from 3.5 to 4.5 g and most of it is bound to hemoglobin in erythrocytes or stored in hepatic and splenic macrophages. Only a tiny percentage of iron (0.1%), which is approximately 0.3 mg, corresponds to extracellular iron, which is bound to plasma TF [54,55].

**Figure 2:**
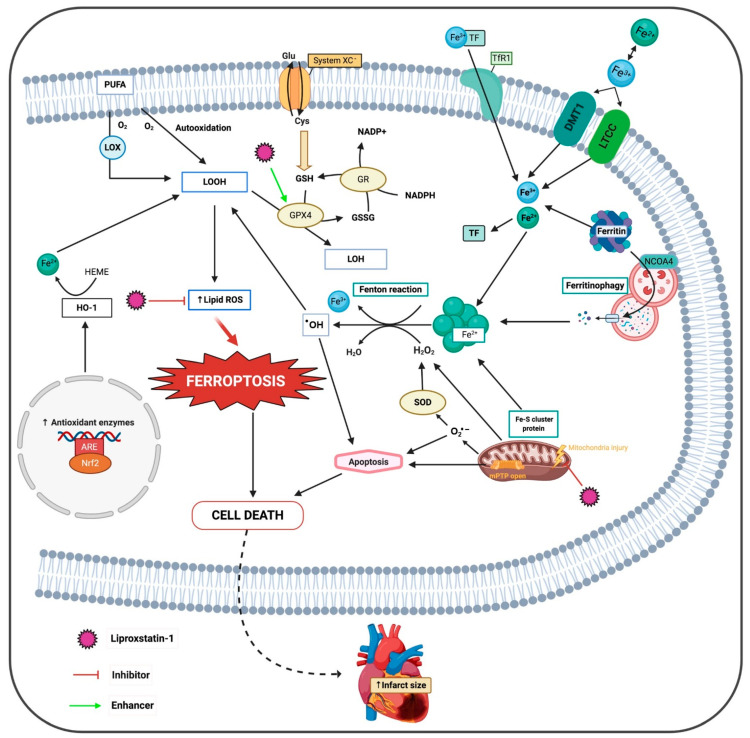
Molecular mechanisms of the deleterious effects of ferroptosis and proposed sites of protective action of liproxstatin-1. ARE, antioxidant response elements; DMT1, divalent metal transporter; Fe^2+^, ferrous iron; Fe^3+^, ferric iron; GPX4, glutathione peroxidase 4; GR, glutathione reductase; HO-1, heme oxygenase 1; H_2_O_2_, hydrogen peroxide; LOH, lipid alcohols; LOOH, lipid hydroperoxides; LOX, lipoxygenase; LTCC, L-type calcium channel; mPTP, mitochondrial permeability transition pore; Nrf2, nuclear factor-erythroid 2-related factor 2; NCOA4, nuclear receptor coactivator 4; O_2_^•−^, superoxide radical; ^•^OH, hydroxyl radical; PUFA, poly-unsaturated fatty acids; SOD, superoxide dismutase; TF, transferrin; TfR1, transferrin receptor.

Iron enters cardiomyocytes mainly as TF, through TfR1, but can also enter as non–TF-bound iron (NTBI) through LTCC, TTCC and the DMT1 (Figure 2). These proteins play a crucial role in the heart under an iron-overloaded condition. Even so, they can be a target to attenuate iron deposition in different organs and, as a consequence, reduce oxidative stress [56]. In normal patients, about 30% of TF is saturated. Thus, under normal iron homeostasis, cardiac supply of iron is provided through highly regulated TF-mediated uptake mechanisms. Otherwise, in pathologies such as hemochromatosis or thalassemia, iron overload occurs and TF becomes saturated. Both conditions can be risk factors for an increased infarct size without reperfusion therapy, because it causes more NTBI to appear in the circulation. NTBI enters the cell, predominantly as ferrous iron, raising the level of labile cardiac iron [57]. Hence, iron metabolism plays an important role in heart IRI because it has different pathways to increase iron.

### 4.2. Pathways of Iron Increase

#### 4.2.1. Myocardial Hemorrhage

Myocardial hemorrhage (MH) is a vital and common event in cardiovascular disease and can cause increased damage due to the deposition of iron in cardiac tissue, especially in ST-segment elevation AMI patients. There are many risk factors for the development of MH after successful reperfusion therapy. MH is more closely associated with adverse outcomes than microvascular obstruction. Patients with this condition experiment adverse left ventricle (LV) remodeling such as a larger LV mass, larger LV volumes, and lower LV ejection fractions [58]. Consistent with this, a study using cardiac magnetic resonance imaging showed that in post-AMI patients that received percutaneous coronary intervention, the presence of MH in all patients with adverse LV remodeling was associated with residual myocardial iron in the surrounding myocardium corresponding to the infarct area [59]. This finding suggests that in myocardial IRI, the ischemia phase may cause lysis of red blood cells, resulting in local accumulation of iron from hemoglobin and heme to be released. This iron accumulation can induce unrestrained inflammation and generate excessive ROS such as ^•^OH or ONOO^−^ [60]. If we are aware that iron accumulation is a critical event for cardiovascular disease, we need to know how this metal participates in pathological processes, not only in cell death like ferroptosis, but also in inflammation or systemic damage.

#### 4.2.2. Degradation of Ferritin

The heart is not the only organ that suffers the consequences of increased iron. Indeed, a high level of iron is involved in the pathology of IRI in a variety of organs. It has been postulated that high FT degradation despite FT synthesis during AMI produces a release of iron into coronary flow [61]. Studies have shown that cardiac function was reduced following ischemia caused by high concentrations of iron that are mobilized into the coronary flow and may contribute to the oxidative damage with myocardial IRI [62]. Moreover, it is not necessary for high levels to produce damage; a study has demonstrated that even mild, non-overloading doses of iron (0.3–12 mg/mL, i.p.) can be detrimental to the heart when an I/R stress is imposed [63]. A high dose of iron caused more than half of a decrease in cardiac work and cardiac output and a reduction in post-ischemic recovery of LV developed pressure, related to an increase in lipid hydroperoxide [20].

#### 4.2.3. Release from Enzymatic Iron-Sulfur Cluster

Iron-sulfur clusters are proteins that have iron-sulfur centers. In this case, during reperfusion, an excess of ROS such as O_2_^•−^ and H_2_O_2_ causes an inbalance in redox homeostasis that damages FT and iron-sulfur cluster-containing enzymes, both iron-containing proteins (Figure 2). Conversion to [3Fe-4S] groups occurs when ROS attack enzymes containing the [4Fe-4S] groups, resulting in labile iron release and inactivation of enzymes [64].

#### 4.2.4. Polyol Pathway

The polyol pathway is an alternative form of glucose metabolism. The result of the activation of this pathway is that the enzyme aldose reductase (AR) irreversibly converts glucose to sorbitol, with the oxidation of its cofactor NADPH to NADP. This continues with the role of another enzyme, sorbitol dehydrogenase (SDH), which converts sorbitol to fructose with the reduction, at the same time, from NAD^+^ to NADH. Therefore, the polyol pathway plays an important role in IRI because it mediates iron-induced oxidative stress [65]. A study showed that SDH activity increased during ischemia, but its inhibition reduced IRI and improved energy homeostasis in hearts. Moreover, on reperfusion, hearts showed better cardiac function than untreated hearts. Therefore, SDH inhibition prevents redox imbalance and ATP depletion, thereby protecting the heart from IRI [66]. AR could contribute to tissue damage by enhancing the generation of ROS catalyzed by iron, especially ^•^OH (due to the Fenton reaction). The lack of this enzyme reduces the increase in TF levels that is observed in tissues subjected to I/R. Indeed, inhibition of the polyol pathway attenuated levels of TF, TfR1, and intracellular iron content in I/R and prevented the increased level of bleomycin-chelatable iron, reducing the production of O_2_^•−^ and malondialdehyde (MDA), a metabolite of lipid peroxidation. The final consequence of polyol pathway inhibition is a smaller final infarct size [67].

#### 4.2.5. Ferritin Heavy Chain

FT is an important protein that maintains iron homeostasis. FT is made up of light and heavy chains. A decrease in FT heavy chain (FTH) is associated with a higher ROS production, which produces cardiac injury and the heart tissue becomes more vulnerable to iron overload-associated ferroptosis. Moreover, a lack of FTH causes a reduction in cardiac Slc7a11 expression, which mediates GSH production [68].

## 5. Ferroptosis

There are two main routes of cell death, apoptotic and non-apoptotic. Apoptosis is a highly controlled cell death process performed by healthy and damaged cells in response to a physiological or pathological stimulus, including I/R events [69]. On the other hand, in non-apoptotic cell death we can highlight ferroptosis, a regulated forms of necrosis that is caused by the accumulation of lipid peroxidation products and ROS derived from iron metabolism, mainly when GSH levels in the cell are depleted or when glutathione peroxidase 4 (GPX4) enzyme is inhibited [70]. GPX4 converts phospholipid hydroperoxides to lipid alcohols using reduced GSH, thus inhibiting ferroptosis [71] (Figure 2).

Furthermore, lipid peroxidation occurs by two main mechanisms, an iron-catalyzed spontaneous peroxyl radical-mediated reaction called autoxidation and an enzyme-mediated process catalyzed by LOXs [72]. LOXs are iron-containing enzymes that catalyze polyunsaturated fatty acid (PUFA) dioxygenation, thus producing lipid hydroperoxides that accumulate in RE [73,74]. These enzymes, primarily 15-LOX, modulate ferroptosis by specifically oxidizing phosphatidylethanolamines (PE), with specificity towards two fatty acyls-arachidonoyl (FAA) and adrenoyl, generating OOH-PE species that act as ferroptotic signals [74]. A recent study has identified a small scaffolding protein inhibitor of kinase cascades, phosphatidylethanolamine-binding protein 1, which complexes with two isoforms of 15-LOX, 15-LO1 and 15-LO2, and changes their substrate affinity from free FAA to FAA-PE to generate OOH-FAA-PE [75]. Recently, new imaging technologies have permitted the detection and visualization of OOH-PE species in ferroptotic cardiomyocytes [76].

Currently, there are two classes of ferroptosis inducers that target specific proteins in the ferroptotic pathway [77], described below.

### 5.1. Class 1 Ferroptosis Inducers

Class 1 ferroptosis inducers such as erastin are one of the main inducers of ferroptosis, which blocks the XC-system, the cystine/glutamate exchanger of the membrane, blocking the entry of cystine into the cell, which is necessary for the synthesis of GSH [78] (Figure 2. Erastin also binds to and inhibits voltage dependent anion channels (VDAC) 2 and VDAC 3, which also leads to cell death [79]. Previous studies demonstrated that although there is no XC-system in the heart, cardiomyocyte death also occurs, but is due to erastin induction [80]. Despite this, the XC-system is encoded by the gene Slc7a11 and its selective overexpression in cardiomyocytes increased GSH levels and prevented cardiac ferroptosis [68].

### 5.2. Class 2 Ferroptosis Inducers

Class 2 ferroptosis inducers such as Ras Selective Lethal 3 directly inhibit GPX4, increase lipid peroxidation, and induce ferroptosis, thus triggering the accumulation of lipid ROS and resulting in cell death [81].

### 5.3. Lysosome and Ferroptosis

There are a variety of organelles that interfere with iron metabolism and are associated with ferroptosis. Lysosome is part of this group because it can modulate iron equilibrium, causing a burst of ROS expression within it, which is attributable to its acid pH and high iron content. Lipid peroxidation may occur in the lysosome membrane due to ROS accumulation and iron overload. Consequently, the lysosomal membrane is permeabilized, causing a greater generation of radicals, cell membrane denaturation, and GSH consumption [82].

Moreover, production of lipid ROS, mitochondrial shrinkage, increased mitochondrial membrane density, and involvement of important transcription factors to produce an antioxidant or pro oxidant response are the main features of ferroptosis. These characteristics make ferroptosis different from other nonapoptotic cell death programs such as apoptosis [83,84]. Studies have revealed that ferroptosis is one of the major drivers of myocardial infarction [9].

### 5.4. Ferroptosis and Relevance of the Cell Membrane

Cell membranes are composed of phosphatidylcholine (PC), which is one of the principal classes of phospholipids. A relevant feature of PC is that it is highly vulnerable to oxidation, forming species related to different pathologies. Oxidized phospholipids (OxPCs) induce cell death only through ferroptosis and not because of apoptosis or necrosis. OxPCs affect the functionality of cardiomyocytes such as changes in Ca^2+^ transients and net cardiomyocyte contraction and can be responsible for reperfusion arrhythmias [85].

### 5.5. Autophagy-Induced Ferroptosis

Ferroptosis is closely related to autophagy, with many molecules involved. Embryonic lethal-abnormal vision like protein1 (ELAVL1) is a protein coding gene that regulates gene expression by stabilizing message RNAs such as TNF-α or VEGF-A and is related to the process of cell death and oxidative stress [86]. ELAVL1 inhibition decreases AMI inflammation responses, so it has a role in myocardial IRI, wherein excessive ROS and inflammatory cytokines were produced with a substantial increase of ELAVL1 [87,88]. Forkhead box C1 (FOXC1) is a transcription factor that has a role in cell growth and survival and also in heart diseases [89].

A recent study [87] showed that FOXC1 transcriptionally-activated ELAVL1 strongly contributes to myocardial IRI through an increase in the autophagic ferroptosis pathway. During I/R, increased ELAVL1 expression produces a deleterious effect on enzyme function and cellular antioxidant capacity because both GSH and GPX4 levels decrease. Nevertheless, ELAVL1 inhibition suppresses ferroptosis and myocardial IRI, restoring GPX4 levels and recovering the viability of myocardial cells, thereby reducing cell injury. The greatest anti-ferroptotic effect of knockdown ELAVL1 is that it inhibits I/R-induced autophagy, thereby protecting against ferroptosis, heart IRI and reducing myocardial infarct size. Moreover, ELAVL1 levels decrease if FOXC1 is knocked down, demonstrating that FOXC1 regulates ELAVL1 expression during I/R. Therefore, the sequence is that ELAVL1 knockdown inhibits ferroptosis and heart IRI and also inhibits autophagy. Autophagy-dependent ferroptosis undoes the effects of ELAVL1 knockdown and contributes to heart IRI, producing overproduction of lipid signaling. This relation between FOXC1 and ELAVL1 and their link to ferroptosis can be a beneficial target against myocardial IRI [87].

### 5.6. Ferroptosis and Necroinflammation

Normally, necrosis is strongly associated with a pro-inflammatory response, where necroinflammation is the immune response to necrosis in a living organism. This can be through an unregulated form such as traumatic necrosis or signaling pathways defined as necroptosis, ferroptosis and pyroptosis, executing necrosis as a regulated process [90]. This cell death pathway, unlike other cell death pathways such as apoptosis, does not present a silent inflammatory response [91]. The evidence of necroinflammation and its relationship with ferroptosis is very poor and much remains to be investigated [92] but recent studies show that ferroptotic cells collapse and release molecular mediators associated with pro-inflammatory damage [93]. Cell markers of the immune system can be detected in various tissues and suggest that ferroptotic cells release immune-stimulating cellular components [94]. So far, most studies in pathologies and genetic models of ferroptosis in vivo have focused on two specific organs: kidney and brain. There is evidence that necroinflammation can occur in the ferroptotic tissue of the kidney, with infiltration of neutrophils and activation of macrophages [95]. We know that IRI in the heart is associated with an inflammatory response and we hypothesize that in the heart tissue, due to the infiltration of inflammatory molecules, a response similar to the kidneys will occur, modulating the final size of the AMI. Hence, it is also necessary to advance studies of ferroptosis in humans.

### 5.7. Ferroptosis and Mitochondria in Cardiomyocytes

The heart has an aerobic metabolism and is dependent on mitochondrial function to obtain energy because cardiomyocytes consume more than 90% of intracellular ATP [96]. For this reason, mitochondria have been studied as a promising target that could be involved in ferroptosis.

During I/R, there is an increase in the oxidation of the mitochondrial matrix and many morphological and metabolic changes occur within the mitochondria. We can highlight rounding and blebbing of the mitochondrial outer membrane. Disorganization and dispersion are also evident [97]. Other morphological changes include a reduction of mitochondria size with condensed mitochondrial membrane densities, reduction of mitochondria cristae, and outer mitochondrial membrane rupture [98]. Some of the changes in the morphological features of mitochondria are not related to other cell death processes, such as necrosis, apoptosis or autophagy, highlighting the relevance of ferroptosis [99].

In fact, there is no consensus on the role of mitochondria in ferroptosis. Nevertheless, recent evidence has demonstrated that mitochondria are organelles involved in the execution of different mechanisms of cell death including ferroptosis, specifically by opening of mPTP and alteration of mitochondrial outer membrane permeabilization [99]. The mitochondrial membrane potential (MMP) is commonly used to evaluate the mitochondrial function, where a loss of the MMP means mitochondrial dysfunction [9,100] and maintaining the MMP is very important to the survival and function of cells that require high-energy, such as myocardiocytes [100].

During mitochondrial-mediated apoptosis, a decrease in MMP was observed before final cell death. This decrease in potential indicates increased permeability of the mitochondrial outer membrane, an important feature of mitochondrial-mediated apoptosis [101]. Although the release of cytochrome C during reperfusion leads to the activation of caspases, interventions exist that inhibit BH3-dependent apoptosis, but do not protect against cell death. This indicates that although apoptosis is activated in I/R, it is not necessary for subsequent cell death, suggesting that other mechanisms may exist to produce cell death [97] such as ferroptosis. Indeed, the role of the mitochondria in this type of cell death is much more proactive than in apoptosis [101].

Gao et al. showed that both mitochondrial tricarboxylic acid (TCA) cycle and mETC play a crucial role in cysteine-deprivation-induced ferroptosis. TCA function supports mETC activity by regulating different complexes in the inner membrane of mitochondria and mETC components are necessary to allow accumulation of lipid ROS, inducing ferroptosis. Indeed, inhibitors of mitochondrial complexes suppressed lipid ROS accumulation and ferroptosis. Moreover, glutaminolysis is associated with I/R injury through ferroptosis and also provides several intermediates into the mitochondrial TCA cycle. Glutamine metabolism has been described as a key event in ferroptosis induction. However, a number of TCA cycle metabolites such as alpha-ketoglutaric acid, succinate, fumarate and malate can recapitulate the function of glutamine in lipid ROS accumulation and ferroptosis induced by cysteine deprivation or XC-system inhibition, indicating that the TCA cycle is required for ferroptosis [101]. It is a known fact that the mitochondria produce ATP using the potential electrochemical proton gradient across the mitochondrial membrane, which is the result of the action of the TCA cycle and mETC. In this sense, it has been described that glutaminolysis, the TCA cycle, and other ferroptosis inducers lead to MMP hyperpolarization, which is associated with ferroptosis and, eventually, may cause the membrane to collapse. Presumably, this hyperpolarization reflects an increase in mETC activity and the subsequent generation and accumulation of lipid ROS [101], where the mitochondrial outer membrane has many PUFAs so the MMP is very sensitive to lipid peroxidation [102]. Thus, glutaminolysis is an essential factor for ferroptosis and its inhibition can protect heart tissue from IRI. Indeed, glutaminolysis inhibitors recover MMP, reduce myocardial infarct size and improve the heart’s function [101,103]. In addition, metabolic changes during I/R initially include a decrease in mitochondrial respiration and an increase in glycolysis. Then, during reperfusion, there is an increase in mitochondrial respiration due to increased oxygen and, consequently, ROS explosion and execution of ferroptosis. The explosion could be due to increased metabolism of the mitochondria because more metabolites are entering [99].

Although mitochondrial function is important, when mETC and glutaminolysis are blocked, ferroptosis will still occur if GPX4 is inhibited. Therefore, it follows that GPX4 has a more direct and effective effect than the other pathways. GPX4 overexpression allows better ATP production, maintains MMP and protects the heart against oxidative damage [102]. In addition, erastin-induced ferroptosis does not require ETC to induce ferroptosis [78]. This indicates that mitochondrial function is dispensable for ferroptosis. Regarding this point, it is important to note that the role of the mitochondria depends on the context. On the one hand, blocking mitochondrial function potently inhibits ferroptosis. On the other hand, GPX4 elimination triggers ferroptosis in cells independent of mitochondria [101]. A recent study demonstrated that OxPCs decrease GPX4 levels and have a direct effect on the mitochondria. During I/R, the release of OxPCs can decrease mitochondrial spare respiratory capacity and, because of this, the ability of mitochondria to respond against oxidative stress is decreased, producing less ATP and, as a consequence, cell death via ferroptosis [85].

Apart from glutaminolysis and the TCA cycle, GSH also plays an important role in the mitochondria. This organelle does not have the CAT enzyme to act on O2^•−^ or the enzymes required for GSH synthesis. GSH acts non enzymatically but is also an essential cofactor for GSH-linked antioxidant enzymes such as GPXs. Under oxidative stress, oxidized glutathione levels increase and the relative proportion between the reduced and the oxidized forms (GSH/GSSG) is considered a marker of oxidative stress. Mitochondrial GSH (mGSH) levels are similar to GSH levels in the cytosol [104]. In terms of mitochondrial oxidative stress balance, mGSH is a critical factor in the control of cell survival/death and regulates factors that influence MMP permeability. Its depletion induces cellular damage and promotes cell death [105,106].

Previously, we identified glutamine as a serological component that is involved in ferroptosis and mitochondria. However, there is another component that is also found in high amounts in the blood, which is TF. This intracellular pathway, including its membrane receptor, is required for ferroptosis. Although this unexpected role of TF is not entirely clear, it is believed that due to its high blood level, a partial cysteine deprivation is sufficient to produce necrosis. Furthermore, under pathological conditions such as I/R, cells are more susceptible to ferroptosis [103].

In summary, ferroptosis can be triggered by different biological pathways. This can be caused by both intracellular and extracellular mechanisms and there are conditions that cause the cell to be more susceptible to this type of cell death such as cysteine deprivation or low levels of GSH [103].

### 5.8. Cell Death Propagation and Ferroptosis

It has been described that ferroptosis importantly involves intercellular interaction, where cell death is transmitted to neighboring cells, spreading in a wave-like manner [95,107], which is characteristic of particular forms of ferroptosis [108], while apoptosis is generally classified as an autonomous cell death that does not induce death in the other cells around it [109].

A recent study describes two different types of ferroptosis based on the spread of cell death. On one hand is cell-autonomous ferroptosis, occurring when GPX4 is inhibited, and on the other is propagative ferroptosis, which is induced by inhibiting the generation of GSH or increasing the concentration of iron [108]. This study also demonstrated that iron and lipid peroxidation are necessary to the propagation of ferroptosis, but not for the cell rupture. However, the speed of the wave propagation is dependent on the lysis of the cell and the mechanism of plasma membrane permeabilization and propagation of cell death must be elucidated [108].

Therefore, we hypothesize that this process could be one of the mechanisms that contribute more to the increase of the infarct size during reperfusion, considering that ferroptosis occurs in heart IRI [9,103,110,111] together with an increase in the concentration of intracellular iron and lipid peroxidation [9,61,62,67,83,112,113]. Therefore, it is necessary to conduct studies focused on heart IRI that can verify the appearance of these waves of ferroptosis in cardiac tissue exposed to I/R, along with its mechanism and importance in the damage process and thus reinforce the idea that ferroptosis is the most important driver of the final infarct size [8,9].

## 6. Does Ferroptosis Occur in the Ischemic Phase or Reperfusion Phase?

Usually, the ischemic phase is overlooked because of the traditional view that exists that ischemia-induced tissue injury and loss of function is an arbitrary consequence of oxygen deprivation, so I/R studies have focused on the reperfusion phase but are of limited translational value [114,115]. Two well recognized biomarkers for ferroptosis are GPX4 and long-chain-fatty-acid-CoA ligase 4 (ACSL4). When ACSL4 and GPX4 proteins levels were studied on ischemic heart, there were no significant changes during different points of ischemia, in addition to no significant changes in iron or MDA contents in the cardiac tissues. As the reperfusion time was extended, levels of ACSL4 protein were gradually elevated, concomitant with a decrease in GPX4 levels in the cardiac tissue, where a gradual increase in iron concentration was also obtained along with increased MDA levels in the myocardium. Therefore, this study finally suggested that ferroptosis occurs during the reperfusion phase in rat hearts that are subjected to an I/R process and not during the ischemic phase [110].

Consistently, these results not only appear in hearts, but also in the intestine and other organs. A previous study [115] suggested that ferroptosis occurs at the early stage of reperfusion. Expression of ACSL4 was induced in ischemic intestines and GPX4 levels were reduced after 45 min of ischemia. These results may sensitize the intestine to ferroptosis reperfusion because this second phase was likely to lead to ferroptosis after 45 min of ischemia. After 15 min of reperfusion, rupture of the outer mitochondrial membrane occurs, and after 30 min the disappearance of mitochondrial cristae was more evident. Moreover, the expression of GPX4 was decreased at 30 min of reperfusion, whereas the expression of cyclooxygenase-2 was increased, as well as 12- (12-HETE) and 15- hydroxyeicosatetraenoic acid (15-HETE), both derived from arachidonic acid. The results showed that ferroptosis was more active 30 min after reperfusion and not during other moments of this phase [115]. Moreover, the polyol pathway and ELAVL1 expression was remarkably elevated in reperfusion [67,87] and in this phase, lysosomes are ready to be released [82]. Therefore, these previous studies finally suggest that ferroptosis occurs during the reperfusion phase in IRI models and not during the ischemic phase, which is very important to take into consideration when developing cardioprotective therapies that inhibit ferroptosis to reduce heart IRI [110].

## 7. Therapies for Myocardial Reperfusion Injury

Interventions that protect the heart from IRI, reducing infarct size, can involve remote ischemic preconditioning and postconditioning [116] and there are many drugs that can reduce myocardial IRI, mainly based on their antioxidant capacity. However, despite the enormous interest in antioxidant vitamins as potential protective agents against the development of human disease, the actual contributions and mechanisms of such compounds remain unclear [6].

### 7.1. Vitamin E

Vitamin E is a fat soluble molecule group considered one of the most potent antioxidants, where α-TOH has been reported as one of the most active forms [117]. This corresponds to an important agent in the prevention of cardiovascular diseases, where a recent study reported that higher α-TOH baseline serum concentration is associated with a decreased risk overall and causes specific mortality for cardiovascular and heart diseases among several other disease groups [118]. On the other hand, the use of vitamin E to reduce damage from cardiac reperfusion ischemia has been shown to present cardioprotection in animal models [119,120,121] and there even could be an association between the beneficial results and the age and gender of the rats subjected to ischemia reperfusion [122].

### 7.2. Ascorbic Acid

Vitamin C or AA is a water-soluble antioxidant agent that acts as a ROS scavenger. It has been studied to demonstrate its cardioprotective effects against cardiac IRI, but the results are debatable. Effects dependent on its route of administration and the concentration of AA given to patients have been described [123], where Davis et al. demonstrated that equal doses of vitamin C orally encapsulated in liposomes generates a higher serum concentration of this antioxidant versus non-encapsulated AA, but less than intravenous AA in human IRI. The three routes of administration produced similar cardioprotective effects against oxidative stress induced by IRI [124]. In addition, Cheng et al. showed that it helps to reduce the inflammatory response in addition to reducing oxidative stress and heart IRI [125].

### 7.3. n-3 Polyunsaturated Fatty Acid

n-3 polyunsaturated fatty acid (n-3 PUFA) of marine origin including alpha-linolenic acid, eicosatetraenoic acid, and docosahexaenoic acid, produce various health effects, and thus are essential nutrients for human health, especially for the cardiovascular system. Concomitantly with IRI, a lipid metabolism disorder occurs in organs such as the heart [126]. Previous studies demonstrated that preconditioning or postconditioning treatment with n-3 PUFA can have anti-inflammatory and antioxidant effects, improving the isolated perfused cardiac function and attenuating mitochondrial damage through the inhibition of NF-κB and induction of Nrf2 [127,128]. Furthermore, other studies demonstrated that n-3 PUFA treatment significantly reduced lipid peroxidation by measuring MDA levels and increased the activity of antioxidant enzymes such as SOD in the myocardium. These facts suggest that n-3 PUFA may improve cardioprotection and prevent IRI due to the attenuation of oxidative damage and its anti-inflammatory activity [125].

### 7.4. Deferoxamine

Deferoxamine (DFO) chelates free iron and is used to treat both acute and chronic iron overload. Although DFO can directly bind and sequester iron from myocardial cells, it will not bind to iron that is already bound to molecules such as TF, FT, hemosiderin, hemoglobin, or cytochromes [129]. The importance of this drug is to act as a chelator for the substrates (Fe^2+^) that participate in the Fenton reaction, preventing ^•^OH formation and subsequent lipid peroxidation.

### 7.5. N-Acetylcysteine

N-Acetylcysteine (NAC) is an acetylated cysteine compound. One of its medical applications is its antioxidant effect due to being a GSH donor. GSH is a molecule of the endogenous antioxidant system, which is used as a substrate by enzymes to reduce other molecules. It also has the ability to act directly as an antioxidant. Therefore, this drug can be used to reinforce antioxidant therapy when GSH levels are decreased during high oxidative states [18,130].

### 7.6. Nuclear Factor Erythroid 2-Related Factor 2

Nrf2 is not an antioxidant but is a transcription factor, and a key regulator of the cellular antioxidant response [131].

Under unstressed condition, low levels of this transcription factor are mainly maintained by Keap1-mediated proteasomal degradation. On the other hand, the protective role of Nfr2, under oxidative stress, is that it plays a role in the protection of our body against stress-induced diseases because it activates several pathways that include nuclear translocation and recruitment of transcriptional coactivators, resulting in the binding between Nrf2 and antioxidant response elements of target genes [132] (Figure 2).

### 7.7. Mechanistic Target of Rapamycin

Several studies demonstrated that the mechanistic target of rapamycin (mTOR) provides cardioprotection against heart IRI [133,134]. In addition to being involved in the regulation of cellular iron uptake and flux by modulating TfR1 [135].

The study by Aoyagi et al. [133] demonstrated that overexpression of cardiac mTOR protected the heart by reducing cellular mortality during the acute phase of in vivo ischemia and preserved cardiac dysfunction caused by heart I/R after 28 days. It also inhibits cardiac fibrosis in adverse LV remodeling and suppresses autophagy in the remote zone after in vivo I/R and suppresses necrosis in ex vivo I/R injury.

### 7.8. Heme Oxygenase-1

Heme oxygenase-1 is a rapidly inducible cytoprotective protein that degrades heme to ferrous iron. A recent study established HO-1 as the major culprit for iron release in doxorubicin (chemotherapeutic drug)-induced cardiotoxicity [9] (Figure 2). Despite this, another study concludes that acute HO-1-mediated cytoprotection extends to the heart and its overexpression improves post-infarction survival and LV remodeling. Moreover, this study shows that HO-1 overexpression attenuates hypertrophy, fibrosis, and oxidant stress, and also promotes neovascularization in the failing heart [136]. Therefore, it is not clear whether HO-1 induction is beneficial or detrimental.

Antioxidants have been used extensively in studies to decrease the infarct size after heart IRI, but these have failed to provide cardioprotection when applied to patients [6]. However, to achieve an effective reduction of the size of the infarct, it is necessary to be able to cover all the pathways that participate in IRI, and one important way of doing this is iron metabolism. Different studies have shown that using more than one antioxidant drug at the same time generates a synergistic effect and thus protects the heart from damage [18,125,137,138].

## 8. Ferroptosis-Based New Strategy to Reduce Infarct Size

As we mentioned before, an important mechanism involved in IRI is ferroptosis. A recent study demonstrated that blocking ferroptosis reduces the severity of myocardial IRI in cardiomyopathy [9] and it has been found that ferroptosis inhibitors can effectively repair I/R-induced cell damage [103]. Drugs capable of inhibiting this pathway include ferrostatin-1, a potent inhibitor of erastin-induced ferroptosis acting as a lipid scavenger [78], and liproxstatin-1 (Lip-1) [70], on which we will focus in this review.

### 8.1. Liproxstatin-1

Several drugs have been used to inhibit ferroptosis in different animal models, but Lip-1 is one of the most promising despites how little is known about it.

#### 8.1.1. Greater Effect of Lip-1 Compared to Others Drugs

Although α-TOH has been described as a potent radical-trapping antioxidant (RTA) that inhibits phospholipid hydroperoxide formation, it is comparatively less potent than Lip-1 [139], where a recent study demonstrated that Lip-1 has a significantly greater ability as a RTA in lipid bilayers and thus inhibits ferroptosis by decreasing lipid autoxidation [140].

Another study compared the effects of Lip-1, DFO and Edavarona in an in vitro model off ferroptotic oligodendrocytes, showing that Lip-1 was more potent and effective than DFO or Edavarona in protecting cells from cell death by ferroptosis, and it was able to act at nanomolar concentrations [141]. However, studies must be performed to extrapolate this efficiency to myocardial cells along with discovering the mechanisms underlying these drugs.

#### 8.1.2. Lipid Peroxide Radical Scavenger

Lip-1 prevents the death of ferroptotic cells by acting as a lipid peroxide scavenger (Figure 2). Lipid ROS production is a distinctive feature of ferroptosis and this is what makes it a special cell death. This mean that ferroptosis inhibitors, such as Lip-1, cannot inhibit other forms of cell death such as necrosis, apoptosis or autophagy and the same occurs with classic inhibitors against ferroptosis [78].

#### 8.1.3. Effect on Mitochondria

Ferroptosis is triggered by extra-mitochondrial lipid peroxidation; therefore, another important feature of ferroptosis is the devolution of mitochondrial structure. Production of ROS from extra-mitochondrial iron-mediated lipid peroxidation results in shrinkage of the mitochondria and rupture of the outer mitochondrial membrane [70,142]. Concomitantly, GPX4 inactivation produces swollen mitochondria and a reduction of cristae [111]. Further, mitochondria are a critical target of myocardial IRI, particularly through VDAC1 regulation and the opening of the mPTP, which can induce cell death [143].

Therefore, post-ischemic Lip-1 administration reduces myocardial infarct size, reduces protein levels of VDAC1, decreases mitochondrial ROS production by the NADH-ubiquinone oxidoreductase (complex I), and protects mitochondrial structural integrity (Figure 2, but it does not affect Ca^2+^-induced mPTP opening [111].

#### 8.1.4. Anti Ferroptotic System Modulation and Production of Antioxidant Enzymes

Lip-1, besides being a lipid ROS scavenger, is an enhancer of the anti-ferroptotic system, where, in a previous study, Feng et al. demonstrated that Lip-1 enhanced the anti-ferroptotic system through the increase of GSH and restoration of GPX4 levels in an I/R model of isolated perfused mice hearts [111]. GPX4 converts phospholipid hydroperoxides to lipid alcohols using GSH, which inhibits ferroptosis by protecting the oxidative damage in the cytosol and nucleus by cytosolic GPX4 and in the mitochondria by mitochondrial GPX4 [144].

Ferroptosis suppressor protein 1 (FSP1) is a another relevant component of the cellular anti ferroptotic system and was initially established as a pro apoptotic gene [145]. Lipid peroxidation products were markedly lower in GPX4-knockout FSP1-overexpressing cells. FSP1 suppresses lipid peroxidation by regenerating antioxidants using NADPH, which reduces coenzyme Q10 (CoQ10) and traps lipophilic radicals. Hence, loss of FSP1 sensitizes to ferroptosis and this study showed that the NADH–FSP1–CoQ10 pathway is a potent suppressor of lipid peroxidation and ferroptosis. Moreover, FSP1 anti ferroptotic function is independent of cellular GSH levels, GPX4 activity, ACSL4 expression and oxidizable fatty acid content [146].

#### 8.1.5. Lip-1 Attenuates Acute Remote Organ Injury after I/R

When I/R causes damage in a particular organ, this may lead to acute injury of remote organs that are not directly related to the focal lesion and this has a critical role in prognosis [147]. However, ferroptosis inhibition by Lip-1 in intestinal I/R mitigated histological injury of the lung and liver. Furthermore, significantly reduced lung edema and decreased myeloperoxidase activity have been observed in the lung and liver [115]. These results indicate that Lip-1 may have a systemic effect, protecting remote organs after I/R.

#### 8.1.6. Use in Humans and Concentrations

There is no evidence to date on the use of Lip-1 in humans. The most recent studies used models like the Langendorff heart model [111] with a Lip-1 concentration of 200 nM or cell lines such as a human renal proximal tubular epithelial cells [70] with different concentrations of Lip-1 (50 nM, 200 nM, 1 μM) or an oligodendroglial cell line derived from a neonatal rat brain with a Lip-1 concentration of 1 μM [141]. Since there is no current consensus on the concentrations of Lip-1 in the different tissues, additional studies will be necessary to deepen the safety of this drug.

### 8.2. Other Ferroptosis Inhibitors

#### 8.2.1. Baicalein

5,6,7-trihydroxyflavone or baicalein is a flavonoid that acts as inhibitor of 12-LOX and 15-LOX [148]. It has been recently shown that baicalein is a potent inhibitor of ferroptosis, but not of apoptosis. This molecule limits erastin-induced iron accumulation and lipid peroxidation, the latter through preventing GPX4 degradation and GSH depletion [149]. Ultimately, several studies have demonstrated that baicalein reduces brain IRI after ischemic stroke suggesting that it has neuroprotective effects [150,151,152].

#### 8.2.2. Mitochondrial-Targeted XJB-5-131

XJB-5-131 is a synthetic antioxidant formed by a nitroxide that acts as a radical scavenger conjugated to a mitochondrial targeting moiety [153]. XJB-5-131 targets mitochondria, providing ROS and electron scavenging capacity [154]. Furthermore, XJB-5-131 inhibits ferroptosis suggesting that intramitochondrial lipid peroxidation has a critical role in this process [155]. In some cases, XJB-5-131 effects have been evaluated on cardiac tissue subjected to IRI in rats, enhancing cardiac tolerance to oxidative stress, improving post-ischemic recovery of cardiac function and inhibiting PTP opening [156,157].

### 8.3. Considerations about Inhibiting Ferroptosis

In this review, we propose that ferroptosis inhibition through Lip-1 can be a therapeutic target to treat IRI, but this process of cell death may be advantageous in other pathologies such as cancer. Indeed, ferroptosis has been described as playing a role in killing tumor cells and suppressing tumor growth [158]. One of the inducers of ferroptosis mentioned above, erastin, can improve the efficacy of chemotherapy when co-administered with chemotherapeutic drugs. Therefore, in clinical settings we have to be aware of the different effects that ferroptosis has not only on the cell, but also on the systemic functioning of the organism because it can be beneficial in some cases and harmful in others [159].

## 9. Conclusions

In summary, oxidative stress and iron metabolism are two of the main pathways of IRI and an example of this injury is percutaneous coronary angioplasty, the gold standard therapy for AMI. However, there is a variety of mechanisms that can increase damage, but these are still unknown. Here, we showed that ferroptosis is a major driver of injury occurring in myocardial infarction and can be a promising target to reduce infarct size. However, we have to be aware of the advantage and disadvantage of ferroptosis according to the pathology in clinical settings. So far, most therapies are focused on pathways that are not directly related to iron metabolism. In light of this, the combination of two or more antioxidants such as DFO (iron chelator) and Lip-1 (scavenger of lipid ROS) or even NAC, AA or vitamin E (antioxidants) may exert synergistic myocardial protective effects because they act in different ways. Another benefit of this combined therapy is that it allows the administration of lower doses. This means that it is not a toxic treatment and an appropriate efficacy can be achieved. In clinical settings, it is difficult to know when a person is going to have an AMI. This is why it is important to act during the reperfusion phase. Since ferroptosis occurs primarily during reperfusion, we hypothesized that the use of Lip-1 before this phase increases antioxidant enzyme activity in myocardial tissue, thereby generating cardioprotection.

## Abbreviations

AA, ascorbic acid; ACSL4, long-chain-fatty-acid-CoA ligase 4; α-TOH, α-tocopherol; AMI, acute myocardial infarction; ANT, adenine nucleotide translocase; AR, aldose reductase; ATP, adenosine triphosphate; CAT, catalase; CoQ10, coenzyme Q10; DFO, deferoxamine; DMT1, divalent metal transporter; ELAVL1, embryonic lethal-abnormal vision like protein1; FAA, fatty acyls-arachidonoyl; Fe^3+^, ferric iron; Fe^2+^, ferrous iron; FOXC1, forkhead box C1; FSP1, Ferroptosis suppressor protein 1; FT, ferritin; FTH, ferritin heavy chain; GPX, glutathione peroxidase; GPX4, glutathione peroxidase 4; GSH, reduced glutathione; HETE, hydroxyeicosatetraenoic acid; HO-1, heme-oxygenase-1; H_2_O_2_, hydrogen peroxide; I/R, ischemia/reperfusion; Keap 1, Kelch-like ECH-associated protein 1; LIP, labile iron pool; Lip-1, liproxstatin-1; LTCC, L-type voltage-dependent Ca2+ channel; LV, left ventricle; MDA, malondialdehyde; mETC, mitochondrial electron transport chain; mGSH, mitochondrial reduced glutathione; MH, myocardial hemorrhage; MMP, mitochondrial membrane potential; mPTP, mitochondrial permeability transition pore; IRI, ischemia-reperfusion injury; mTOR, mechanistic target of rapamycin; NAC, N-acetylcysteine; NADPH, reduced nicotinamide adenine dinucleotide phosphate; NHE-1, Na^+^/H^+^ exchanger-1; NF-κB, nuclear factor kappa B; NO^•^, nitric oxide radical; NOO^•^, nitrogen dioxide radical; NOSs, NO synthases; Nrf2, nuclear factor-erythroid 2-related factor 2; NTBI, non–TF-bound iron; O_2_^•−^, superoxide; ^•^OH, hydroxyl radical; ONOO^‒^, peroxynitrite anion; OxPCs, oxidized phospholipids; PE, phosphatidylethanolamines; PC, phosphatidylcholine; PUFA, polyunsaturated fatty acid; ROS, reactive oxygen species; RNS, reactive nitrogen species; RTA, radical-trapping antioxidant; SDH, sorbitol dehydrogenase; SOD, superoxide dismutase; TCA, tricarboxylic acid; TF, transferrin; TfR1, transferrin receptor; TNF-α, tumor necrosis factor; TTCC, T-type voltage-dependent Ca^2+^ channel; uncNOS, uncoupled nictric oxide synthase; VDAC, voltage dependent anion-selective channel; VEGF, vascular endothelial growth factor; XC- system, cystine/glutamate transporter; XO, xanthine oxidase.
